# Increasing Incidence of Proteus- Evaluating the ‘Big Three’ Carbapenem-Resistant Enterobacterales in Tennessee and the Southeast Region

**DOI:** 10.1017/ash.2025.423

**Published:** 2025-09-24

**Authors:** Lauren Daniel, Priscilla Pineda, Darryl Nevels, Melphine Harriott, Erin Hitchingham, Michael Norris

**Affiliations:** 1 Tennessee Department of Health; 2 Tennessee Department of Health; 3 Tennessee Department of Health; 4 TN Department of Health HAI/AR Program; 5 Tennessee Department of Health; 6 State of Tennessee Department of Health

## Abstract

**Background:** According to the Centers for Disease Control and Prevention (CDC), carbapenem-resistant Enterobacterales (CRE) are an urgent public health threat. The CDC states the most common or ‘Big Three’ CRE are Escherichia coli, Enterobacter species, and Klebsiella species. States look at the ‘Big Three’ for guidance when setting reportable condition criteria for CRE. Evaluating trends of the non-‘Big Three’ genera is critical to ensure surveillance efforts are focused on priority targets. Thus, CRE genera trends were evaluated to verify the fitness of CRE surveillance reporting recommendations. **Method:** The Antimicrobial Resistance Laboratory Network (ARLN) Southeast region (SER) includes Alabama, Florida, Georgia, Louisiana, Mississippi, Tennessee, and Puerto Rico. All CRE is reportable in Tennessee (TN) and isolate submission is required to ARLN. Other jurisdictions submit CRE to the TN regional lab. Submitted CRE cases to ARLN from 2018 – 2023 were analyzed. CRE cases were defined as an Enterobacterales organism resistant to one or more carbapenem, excluding imipenem for Proteus sp., Providencia sp., or Morganella sp. due to intrinsic resistance. Data was cleaned in SAS v9.4 to provide descriptive CRE statistics. **Result:** The top three genera for TN fluctuate between Enterobacter sp., Klebsiella sp., Proteus sp., and Escherichia sp. In 2022, Proteus sp. (n=132) had twice the incidence of Escherichia sp. (n=65) in TN. There was an overall increasing trend of Proteus sp. from 2018 – 2023. The largest increase of Proteus sp. in TN was seen between 2018 (n=23) and 2021 (n=183). However, the prevalence sharply decreased between 2021 (n=183) and 2023 (n=12). Proteus sp. was 17% (n=627) of all CRE cases (n=3625) in TN from 2018 – 2023, while the “Big Three” was 72% (n=2628). In contrast, Proteus sp. was only 3% (n=35) of all CRE cases (n=1400) in the SER excluding TN from 2018 – 2023, compared to 89% (n=1250) for the “Big Three”. **Conclusion:** CRE surveillance identified an increased overall prevalence of Proteus sp. in TN between 2018 and 2022 despite not being included in the ‘Big Three’. While there was a large increase of Proteus sp. observed in 2021, the increase was limited to TN, and the subsequent decline suggests this is an outlier. Jurisdictions outside of TN often only submit Carbapenemase-producing CRE to ARLN as not all jurisdictions have CRE as a reportable condition. Results of this analysis suggest the SER should continue to monitor CRE and Proteus sp. to note if there is an increasing overall trend to better inform isolate submission strategies.

